# An Activatable Lanthanide Luminescent Probe for Time‐Gated Detection of Nitroreductase in Live Bacteria

**DOI:** 10.1002/anie.202002391

**Published:** 2020-04-16

**Authors:** Benjamin Brennecke, Qinghua Wang, Qingyang Zhang, Hai‐Yu Hu, Marc Nazaré

**Affiliations:** ^1^ Medicinal Chemistry Leibniz-Forschungsinstitut für Molekulare Pharmakologie 13125 Berlin Germany; ^2^ State Key Laboratory of Bioactive Substances and Function of Natural Medicine Institute of Materia Medica Peking Union Medical College and Chinese Academy of Medical Sciences Beijing 100050 China

**Keywords:** bacterial imaging, enzymes, lanthanides, luminescence, nitroreductase

## Abstract

Herein we report the development of a turn‐on lanthanide luminescent probe for time‐gated detection of nitroreductases (NTRs) in live bacteria. The probe is activated through NTR‐induced formation of the sensitizing carbostyril antenna and resulting energy transfer to the lanthanide center. This novel NTR‐responsive trigger is virtually non‐fluorescent in its inactivated form and features a large signal increase upon activation. We show that the probe is capable of selectively sensing NTR in lysates as well as in live bacteria of the ESKAPE family which are clinically highly relevant multiresistant pathogens responsible for the majority of hospital infections. The results suggest that our probe could be used to develop diagnostic tools for bacterial infections.

## Graphical abstract

**Illuminating pathogens**: A terbium‐based luminescent turn‐on probe was developed capable of tracing nitroreductase in live bacteria using fluorescence lifetime imaging. The probe thus could be used as a new tool for the imaging of bacterial infections.

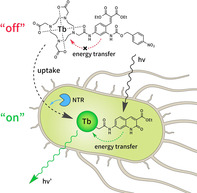

Optical imaging tools are an indispensable analytical modality for the investigation and understanding of complex biological processes ranging from molecular level to whole organisms. Small‐molecule fluorophores are of particular relevance as their properties are readily tuned and modified to incorporate specific functions for monitoring and determining biological activity in situ. In this context, luminescent lanthanide complexes have attracted considerable attention since they have several inherent advantages over conventional organic fluorophores, making them highly promising tools for future medical applications, such as biosensing and bioimaging.^1^ These features include a large Stokes shift avoiding spectral cross‐talk, long‐lived emission allowing for time‐gated detection without interfering background fluorescence, high photostability as well as simple tuning of the emission wavelengths from visible to near infrared and lanthanide‐dependent fingerprint‐like emission bands allowing for ratiometric analyses.^2^

As a result of Laporte‐forbidden f–f transitions and the resulting low extinction coefficient, effective excitation of the lanthanide requires a sensitizing antenna in the form of an organic chromophore enabling energy transfer.^2^ Elegant examples for responsive lanthanide luminescent probes based on the analyte‐induced modulation of the antenna or the chelation properties of the lanthanide have been designed for sensing small inorganic species, such as ^1^O_2_, HCO_3_^−^, and pH changes in cells.^3^ However, tracking intracellular enzymatic activity with such probes still poses a major obstacle and, despite recent pioneering efforts,^4^ is often hampered by synthetic hurdles and suboptimal physicochemical properties, such as low permeability and weak emission.^5^

As part of our program on DOTA‐based fluorescent probes^6^ we were in particular interested in investigating activatable lanthanide luminescent probes for the detection of nitroreductase (NTR) in bacteria.^7^ NTRs are a family of flavin‐containing bacterial enzymes that are able to reduce nitro functional groups and other nitrogen‐containing functionalities in the presence of NADH or NADPH.^8^ The occurrence of NTRs in bacterial pathogens, such as in members of the ESKAPE family (*Enterococcus faecium*, *Staphylococcus aureus*, *Klebsiella pneumoniae*, *Acinetobacter baumannii*, *Pseudomonas aeruginosa*, and *Enterobacter* species), which are responsible for the majority of hospital infections with multiresistant strains escaping the standard antibiotics treatment, makes this enzyme family a highly relevant diagnostic target for the detection of bacterial infections.^9^ Although probes capable of detecting NTRs in bacteria have been developed, the exploitation of NTRs as diagnostic markers for bacterial infections remains scarce.7b, 10 Moreover, probes utilizing the advantageous features of lanthanide luminescence have not been investigated so far.

Herein, we present the development of the first enzyme‐triggered lanthanide NTR probe which 1) is based on a novel responsive antenna‐forming element, 2) is highly sensitive, selective and stable, 3) gives a fluorescence readout over virtually no background signal and 4) is capable of selectively tracking NTR activity in live bacteria.

The overall concept for the design of our NTR‐responsive lanthanide probe was to employ a non‐sensitizing caged antenna precursor which, upon interaction with the enzyme, would initiate a self‐immolative fragmentation cascade thereby forming the antenna enabling energy transfer to the lanthanide center (Scheme 1).^4^, ^11^ Constructing a stably caged, non‐sensitizing NTR‐responsive antenna precursor as key element for our probe proved to be crucial. Although carbostyril antennas are known to be efficient sensitizers and might offer distinct advantages over their corresponding coumarin analogues, such as a more efficient energy transfer and higher quantum yields,^12^ a caging strategy for carbostyril precursors has so far not been investigated. We designed our probe in such a way that antenna formation occurs upon NTR‐mediated reduction of the nitro group of the *para*‐nitro benzylcarbamate to the corresponding aniline, initiating fragmentation to the iminoquinone methide (Scheme 1). Subsequent lactamization through intramolecular attack of the liberated amine at the ester should then yield the corresponding carbostyril as an effective sensitizer of the terbium center. Additionally, by directly attaching the precursor to one carboxylic acid arm of the DOTA scaffold we aimed for a short distance between carbostyril antenna and lanthanide center as it is reported as a critical factor for an effective sensitization of the lanthanide.12d

**Scheme 1:**
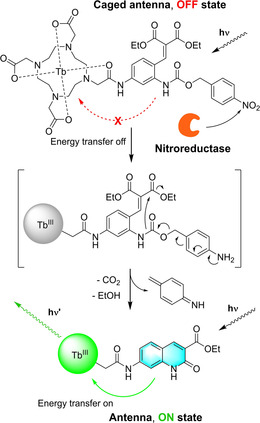
Design rationale and mode of action of the carbostyril‐based NTR activatable luminescent lanthanide probe.

The synthesis of both the antenna **6** as well as the caged antenna precursor **8** started from 2‐bromo‐4‐nitrobenzoic acid (**1**, Scheme 2, left) which was converted into the aminoester **2**. Reduction to the alcohol and Boc protection of the amine furnished **3**, which was further converted into the corresponding benzaldehyde **4** serving as important intermediate to access both **6** and **8**. For the subsequent Buchwald‐Hartwig type coupling with either *tert*‐butyl carbamate or 4‐nitrobenzyl carbamate, XantPhos Pd G3 was employed as a palladacycle precatalyst.^13^ This reaction served as key step in the synthesis of both the carbostyril **6** and the caged antenna precursor **8** and allowed us to selectively introduce and discriminate between the two functionalized amines at C‐2 and C‐4 in compound **7**. Furthermore, this strategy enables for simple functionalization at C‐2 and thus constitutes a robust and general modular approach for the synthesis of similar types of analyte‐responsive triggers. Condensation with diethyl malonate^14^ and subsequent Boc deprotection yielded the caged antenna **8**, or, in case of **5**, induced cyclization to the carbostyril antenna **6**. Attachment of the caged antenna and the carbostyril to the DOTA scaffold was accomplished by reaction of the amine intermediates **6** and **8** with bromoacetyl bromide and subsequent alkylation of the free amine of *tert*‐butyl protected DO3A (Scheme 2, right). Acidic cleavage of the *tert*‐butyl groups furnished conjugates **9** and **11**. Terbium(III) complexation was accomplished by reaction with TbCl_3_ in ethanol at slightly elevated temperatures with small amounts of water to facilitate dissolution. The application of such mild conditions ensured that the ethyl esters of both **10** and **12** remained intact during the course of the reaction.

**Scheme 2:**
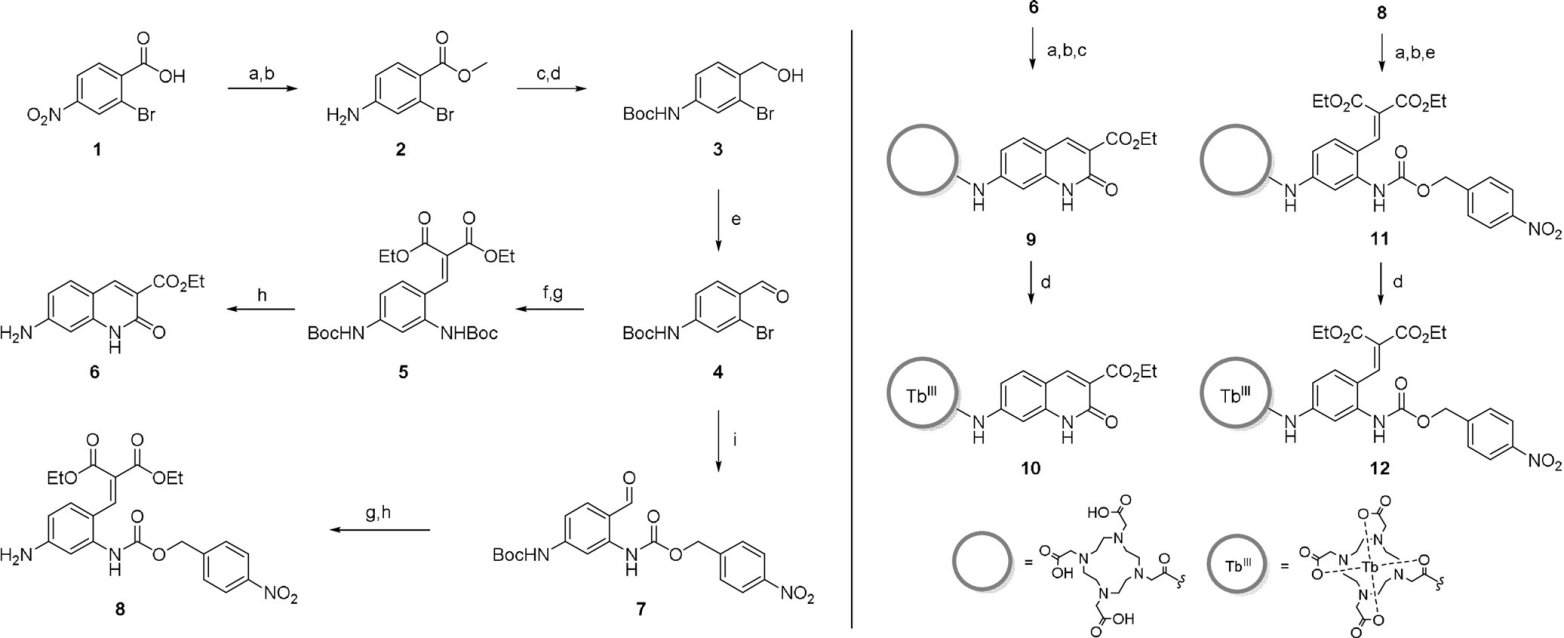
Left: Synthesis of Antenna **6** and caged antenna **8**. a) SOCl_2_, MeOH, reflux, 18 h, 92 %. b) SnCl_2_ 2H_2_O, EtOH, reflux, 4 h, 94 %. c) LiAlH_4_, THF, 0 °C, 4 h, 67 %. d) Boc_2_O, DIPEA, THF, RT, 4 days, 82 %. e) Dess–Martin‐reagent, CH_2_Cl_2_, RT., 18 h, 76 %. f) *tert*‐Butyl carbamate, XantPhos Pd G3, Cs_2_CO_3_, dioxane, 100 °C, 18 h, 69 %. g) Diethyl malonate, TiCl_4_, pyridine, THF, 0 °C–RT, 18 h, **5**: 32 % **8**: 81 %. h) TFA/DCM 1:1, RT, 30 min, **6**: quant., **8**: 64 %. i) 4‐Nitrobenzyl carbamate, XantPhos Pd G3, Cs_2_CO_3_, dioxane, 100 °C, 4 h, 88 %. Right: Attachment of **6** and **8** to the DOTA scaffold leading to carbostyril reference probe **10** and NTR probe **12**. a) Bromoacetyl bromide, K_2_CO_3_, CH_3_CN, 0 °C, 2 h. b) Tris(*t*Bu)DO3A, K_2_CO_3_, CH_3_CN, RT, 18 h. c) TFA/DCM 1:1, RT, 18 h, 14 % over 3 steps. d) TbCl_3_, H_2_O, EtOH, 45 °C, 24 h, 10: 50 %, 12: 40 %. e) 4 n HCl in dioxane, RT, 18 h, 47 % over 3 steps.

Figure 1 A shows the absorption, excitation and fluorescence spectra of the carbostyril probe **10** and the fluorescence spectrum of the caged precursor **12**. As expected, the caged probe **12** does not exhibit any fluorescence at all, also showing that the caged antenna **8** is not able to sensitize the terbium(III) complex.

**Figure 1:**
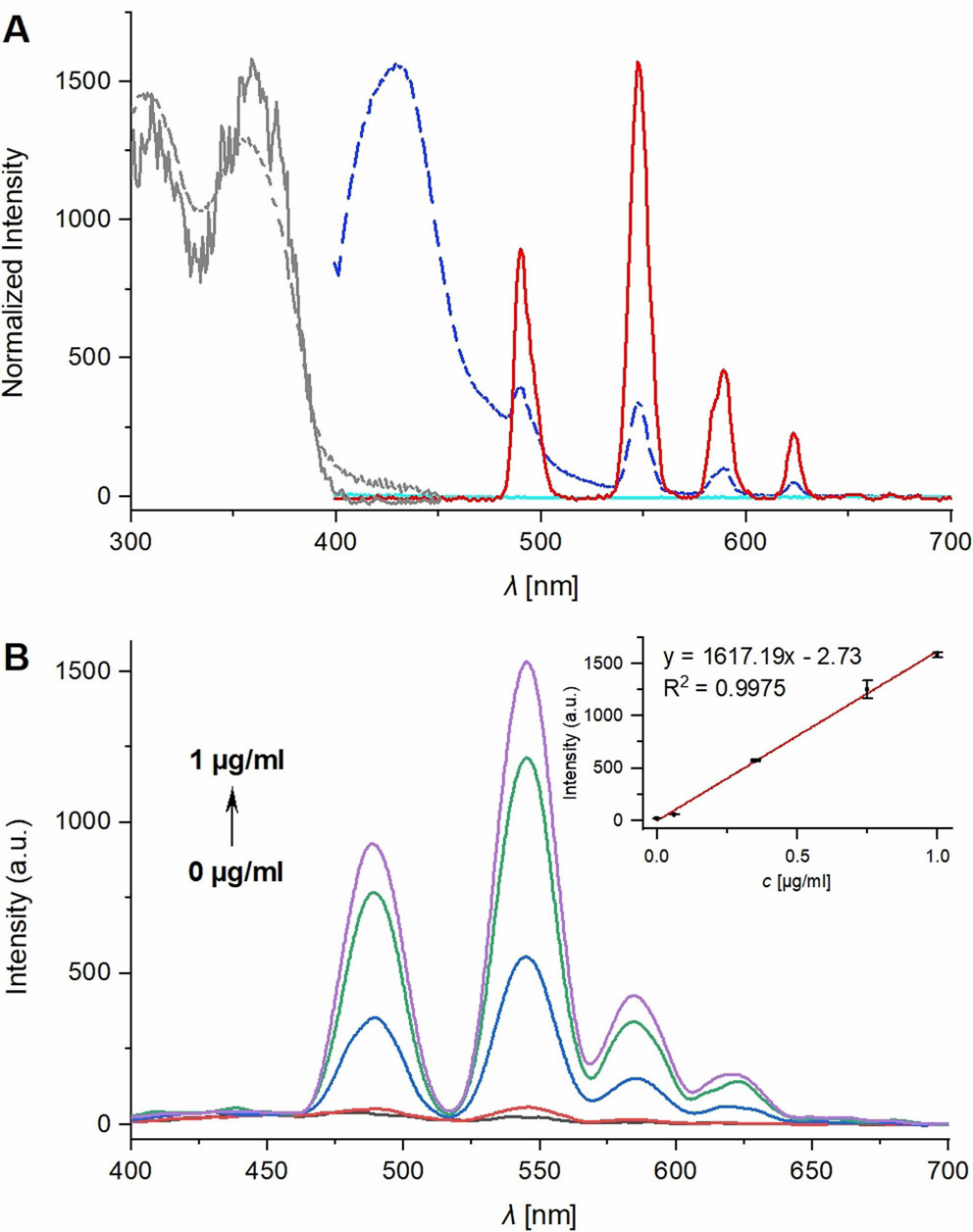
A) Normalized absorption (dotted gray), excitation (monitored at 545 nm, gray) and fluorescence spectra of reference **10** (steady‐state, dark blue and with 50 μs delay, red) and fluorescence spectrum of probe **12** (steady‐state, light blue), each 20 μm in PBS pH 7.4. B) Gated emission spectra of probe **12** (20 μm) upon titration with different concentrations of NTR (0, 0.0625, 0.35, 0.75 and 1.0 μg mL^−1^) in TRIS buffer (50 mm, pH 7.4) at 37 °C for 2 h. Inset: Linear correlation between NTR concentration and emission intensity at 550 nm, 50 μs delay, *λ*_ex_=355 nm. Results for (B) representative of two independent experiments.

While the steady‐state emission of reference probe **10**, constituting the probe's activated state, contains the strong antenna fluorescence signal at 435 nm, under time‐gated conditions (50 μs delay) only the terbium emission bands are visible. The absorption and excitation spectrum are similar, validating Tb sensitization by the antenna. The total luminescence quantum yield of complex **10** was determined to be 1.67 %, which is amongst the best values for turn‐on luminescent probes featuring analyte‐triggered antenna formation.^4^, ^11^ The lifetime of the complex was determined to be 68 μs, which significantly increased to 580 μs upon deoxygenation of the sample solution, indicating energy back transfer from the Tb excited state to the carbostyril triplet state (see Figure S4, Table S1 in the Supporting Information).

Next, we investigated the probe's response to NTR and its ability for sensing NTR in a biochemical setting. Figure 1 B shows the gated emission spectra of probe **12** in presence of increasing amounts of NTR. While in absence of the activating enzyme no fluorescence can be detected, the emission intensities gradually increase with rising concentrations of NTR with a 126‐fold signal increase at 550 nm at 1 μg mL^−1^ NTR. A linear correlation was obtained over a broad range of enzyme concentration versus emission intensity at 550 nm. Furthermore, the detection limit (3*σ*/*k*)^15^ was calculated to be as low as 4.4 ng mL^−1^. The apparent kinetic parameters *K*_M_ and *v*_max_ for the activation of our probe by NTR (18.4 μm and 0.027 μm min^−1^, respectively, see Figures S4 and S5) are in good agreement with previously reported values for other NTR probes.10d LC/MS studies further substantiated the underlying activation mechanism of caged probe **12** by NTR suggested in Scheme 1 by confirming the time‐dependent conversion to its activated state **10** as well as revealing the appearance of a carbamic acid intermediate resulting from the fragmentation (see Figure S6). Evidence for the NTR‐specificity of our probe emerged from the addition of ascending concentrations of the NTR‐inhibitor dicoumarin^16^ where we observed a concentration dependent gradual reduction of the luminescence, confirming that activation of our probe arises from the NTR enzyme‐catalyzed reduction reaction (see Figures S9, S10).

Subsequently, we determined the specificity and stability of our probe **12** against other commonly present bioanalytes in the bacterial cellular environment, as it is crucial to exclude any interfering factors for an accurate determination of NTR activity when applied in bacteria. As shown in Figure 2 A, exclusively NTR triggered a response while the probe remained virtually silent in presence of other species including high concentrations of reductive thiols such as glutathione or dithiothreitol, indicating high specificity for NTR detection.

**Figure 2:**
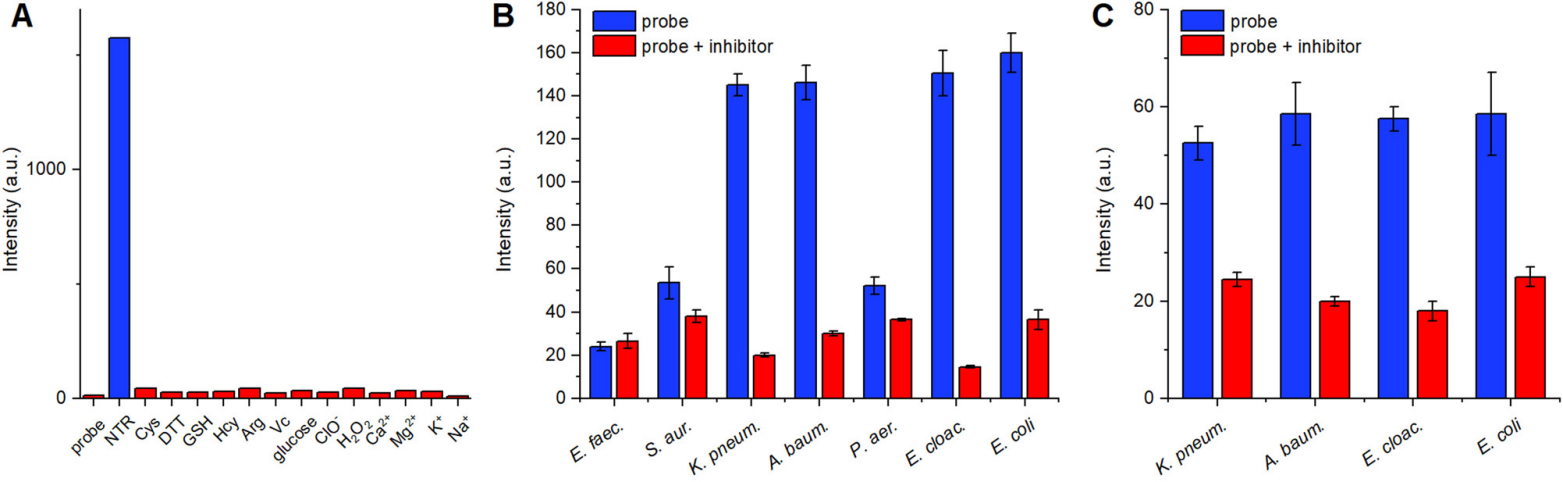
A) Fluorescence responses of probe **12** (20 μm) to various species after 2 h incubation: NTR (1 μg mL^−1^), Cys (1 mm), DTT (1 mm), GSH (1 mm), Hcy (1 mm), Arg (1 mm), Vc (1 mm), glucose (10 mm), ClO^−^ (10 mm), H_2_O_2_ (10 mm), CaCl_2_ (2.5 mm), MgCl_2_ (2.5 mm), KCl (10 mm), and NaCl (10 mm). B) Emission intensities of ESKAPE cell lysates incubated with probe **12** (20 μm) in the absence or presence of the NTR inhibitor dicoumarin (0.1 m) for 2 h. C) Fluorescence intensities of live *K*. *pneumoniae*, *A. baumannii*, *E. cloacae*, and *E. coli* cells incubated with probe **12** (20 μm) in the absence or presence of the NTR inhibitor dicoumarin (0.1 m) for 4 h. All spectra were acquired at 37 °C in 0.05 m Tris buffer (pH 7.4). *λ*_ex_/*λ*_em_=355/550 nm, 50 μs delay. Results representative of two independent experiments.

We then examined the ability of our probe to detect NTR in bacterial lysates. Six bacterial strains of the clinically relevant ESKAPE panel as well as *E. coli* were selected. The bacteria were lysed by sonication and the lysate was incubated with probe **12** with or without dicoumarin inhibitor for 2 h before recording the emission signal at 550 nm. As shown in Figure 2 B, our probe was strongly activated in *K. pneumoniae*, *A. baumannii*, *E. cloacae*, and *E. coli* while the activation was less pronounced in the other three strains. Co‐incubation with the NTR‐inhibitor significantly reduced probe activation in *K. pneumonia, A. baumannii*, *E. cloacae*, and *E. coli*, indicating selective intracellular activation by NTR, while showing little to no effect in the other three. These differences could be attributed to different expression levels as well as species differences of NTR enzymes amongst the bacterial strains, as sequence alignments of those enzymes showed low conservation amongst the bacterial strains as proposed in preceding work.10d

We advanced to test whether we could detect NTR in live bacteria. We were pleased to get similar results in *K. pneumoniae*, *A. baumannii*, *E. cloacae*, and *E. coli* to those obtained with the lysates. While probe **12** was highly activated in all four strains, co‐incubation with NTR‐inhibitor dicoumarin led to a drastic decrease in activation (Figure 2 C). The activation of probe **12** was further investigated by fluorescence lifetime imaging (FLIM) in live *E. coli*. Figure 3 shows the long‐lived emission fluorescence signals with an average lifetime of 31 μs. These results suggest that probe **12** was readily taken up by the bacterial cells and triggered by intracellular NTRs. To our knowledge, this is the first example of FLIM applied to image enzymatic activity in live bacteria with lanthanide luminescent probes. Interestingly, we observed that in contrast to caged probe **12**, which was readily taken up by bacterial cells, the activated reference **10** was not able to permeate into and label bacterial cells (Figure S11). Moreover, in the FLIM experiments activated probe **10** remained localized in the bacterial intracellular compartments and was not cleared to the extracellular medium, for example, by an efflux mechanism, as observed for a previous enzyme‐activatable lanthanide probe.^4^ Collectively, these findings suggest a synergistically enhanced localization, detection specificity, and contrast due to intracellular enrichment of the activated probe **10**.

**Figure 3:**
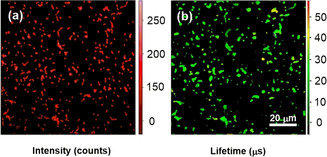
Fluorescence lifetime imaging of live *E. coli* bacteria incubated with probe **12** (20 μm) for 4 h at 37 °C. a) Fluorescence intensity image, b) lifetime map. *λ*_ex_=375 nm, fluorescence intensities and lifetimes were collected through a 641/75 nm long‐pass edge filter.

In summary, we have developed the first luminescent turn‐on probe for the highly selective and sensitive detection of nitroreductase. Installation of a new type of analyte‐responsive carbostyril forming switch was enabled through the key Buchwald–Hartwig type synthetic transformation providing a robust modular approach for the facile access to similar probes. Owing to its effective intracellular enrichment, our probe enables the simple detection and imaging of nitroreductase activity in live bacteria that belong to potentially multiresistant strains of the clinically highly relevant ESKAPE panel. This permitted the first demonstration of fluorescence lifetime imaging (FLIM) to trace enzymatic activity in live bacteria with lanthanide luminescent probes. Taken together these features illustrate that this type of probe concept is an attractive option for future analytical applications in medical diagnostics.

## Conflict of interest

The authors declare no conflict of interest.

## Supporting information

As a service to our authors and readers, this journal provides supporting information supplied by the authors. Such materials are peer reviewed and may be re‐organized for online delivery, but are not copy‐edited or typeset. Technical support issues arising from supporting information (other than missing files) should be addressed to the authors.

Supplementary
