# Supplementary material for: An Activatable Lanthanide Luminescent Probe for Time‐Gated Detection of Nitroreductase in Live Bacteria
Source: Angew Chem Int Ed Engl. 2020 Apr 16;59(22):8512–6. doi: 10.1002/anie.202002391 (PMC7317344; doi:10.1002/anie.202002391)
Supplement: Supplementary file 1 — Supplementary [file ANIE-59-8512-s001.pdf]

Angewandte Chemie
Eine Zeitschrift der Gesellschaft Deutscher Chemiker

Supporting Information

An Activatable Lanthanide Luminescent Probe for Time-Gated Detection of Nitroreductase in Live Bacteria

Benjamin Brennecke, Qinghua Wang, Qingyang Zhang, Hai-Yu Hu, and Marc Nazaré**

anie_202002391_sm_miscellaneous_information.pdf

Table of Contents

Experimental Procedures **4**

Preparative Methods 4

Analytical Methods 4

UV-VIS absorption and fluorescence spectra 4

Determination of luminescent lifetimes 4

Determination of luminescence quantum yield 4

Measurements of NTR activity in bacterial lysates and live bacteria 4

Synthetic Procedures 6

Probe Characterization **12**

Absorption/Excitation/Emission Spectra 12

Lifetime Measurements 13

Determination of kinetic parameters 14

LC/MS activation studies 15

Effects of pH and temperature 16

Detection Limit 16

Inhibition Studies 17

Probe and reference uptake in live bacteria 18

Author Contributions **18**

Experimental Procedures

Preparative Methods

Reactions requiring anhydrous conditions were carried out in dry solvents stored over molecular sieves (THF, DCM, toluene, pyridine, DMF from Sigma Aldrich) which were used as received. These moisture- and air-sensitive reactions were conducted under nitrogen using Schlenk-technique.

Purification of compounds was performed by flash chromatography on silica using a Biotage Isolera One apparatus or by HPLC using a Waters system equipped with a Waters 2489 UV/visible detector, a Waters 2545 Binary Gradient Module and a Nucleodur 100-7 C18ec column from Macherey-Nagel. Water/acetonitrile with 0.1% TFA was used as the solvent system, with one of the following methods. **Method A:** 15-45% CH_3CN 0-18 min, then 45-100% 18-23 min, 100% 23-28 min. **Method B:** 5-35% CH_3CN 0-24 min, 35-100% 24-30 min, 100% 30-34 min. **Method C:** 5-50% CH_3CN 0-35 min, 50-100% 35-40 min, 100% 40-43 min.

Analytical Methods

NMR spectra were acquired on devices from the company Bruker (AV 300, AV 600). All ^{13}C-NMR-spectra were recorded with ^{1}H-broad-band decoupling. Calibration of the chemical shift was conducted using the solvent residual signals. Numbering of the denoted compounds arises from the IUPAC nomenclature. Mass spectra were recorded with an Agilent 1260 liquid chromatography coupled accurate mass time-of-flight 6230 detector. Standard analysis of reaction time courses were conducted with an Agilent 1260 infinity liquid chromatography coupled quadrupole mass spectrometer 6120 detector.

UV-VIS absorption and fluorescence spectra

The concentration of DMSO stock solution of control probe **10** (10 mM) and nitroreductase probe **12** (10 mM) were diluted to 20 µM in the according buffer. The UV-Visible spectra were recorded using a Tecan Spark™ 10M Multimode Microplate Reader, wavelength interval: 5.0 nm. Fluorescence spectroscopic studies were also performed at the excitation wavelength of 355 nm, wavelength interval: 5.0 nm.

Determination of luminescent lifetimes

The luminescence emission decays of the compounds (20 µM in Tris-HCl buffer, pH 7.4) were recorded with an Edinburgh photonics FLS980 with the excitation wavelength set to 355 nm and the emission monitored at 550 nm. The luminescence emission was fitted to a single exponential decay as the most suitable fit according to R^2 and χ^2 to obtain the lifetime constants. Data is representative of a single experiment.

Determination of luminescence quantum yield

The total quantum yield of the complex was determined in aq. PBS pH 7.4 with an integrating sphere using an Edinburgh photonics FLS980. The quantum yield was calculated using the direct excitation method, consecutively exciting a reference sample solution containing aq. PBS and a probe containing sample solution set to an optical density of around 0.1 at the excitation wavelength. All emissions were corrected by the wavelength sensitivity of the spectrometer. The total quantum yield was then calculated according to the equation $\Phi_{tot} = (E_s-E_r)/(S_r-S_s)$, where E_s and E_r correspond to the integrated emission peaks of the sample and the reference, and S_s and S_r correspond to the integrated scatter peaks of the sample and the reference, respectively.[1] Data is representative of a single experiment.

Measurements of NTR activity in bacterial lysates and live bacteria

Nitroreductase (≥100 units/mg) from Escherichia coli and reduced nicotinamide adenine dinucleotide (NADH) were purchased from Sigma-Aldrich. The bacterial strains *Escherichia coli* (*E. coli*, ATCC 25922), *Staphylococcus aureus* (*S. aureus*, ATCC 29213), *Klebsiella pneumoniae* (*K. pneumonia*, ATCC 700603), *Pseudomonas aeruginosa* (*P. aeruginosa*, ATCC 27853) were purchased from American Type Culture Collection (ATCC), USA. *Enterococcus faecium* (*E. faecium*, CICC 10840) and *Acinetobacter baumannii* (*A.*

baumannii, CICC 22933) were purchased from China Center of Industrial Culture Collection, CICC®. OD values were recorded in a 10 mm path quartz cell on a Metash UV-5100B spectrometer.

NTR activity in bacterial lysates. The six bacterial strains were cultured for 12 h in Luria-Bertani (LB) medium at 37 °C, then harvested and washed twice with Tris buffer (pH 7.4). The washed cells were resuspended in Tris buffer with an OD600 of 10.0. The cell suspension was then aliquoted and lysed by sonication. The lysates were then treated with 20 μM of nitroreductase probe **12** for 2 h with or without dicoumarin (0.1 mM) and fluorescence of the reaction solutions was measured using a Tecan Spark™ 10M Multimode Microplate Reader. ($\lambda_{ex}/\lambda_{em}$ = 355/550 nm)

NTR activity in live bacteria. The three bacterial strains were cultured for 12 h in Luria-Bertani (LB) medium at 37 °C, and then harvested and washed twice with Tris buffer (pH 7.4). The washed cells were resuspended in Tris buffer with an OD600 of 2.0. The cell suspension was then aliquoted and treated with 20 μM of nitroreductase probe **12** for 4 h and 12 h with or without dicoumarin (0.1 mM). Fluorescence of the bacterial solutions was measured using a Tecan Spark™ 10M Multimode Microplate Reader ($\lambda_{ex}/\lambda_{em}$ = 355/550 nm).

Uptake of probe 12 and reference 10 in live bacteria. *E. coli*, *K. pneumoniae, A. baumannii* and *E. cloacae* cells were cultured for 12 h in Luria-Bertani (LB) medium at 37 °C, and then harvested and washed twice with Tris buffer (pH 7.4). The washed cells were resuspended in Tris buffer with an OD_{600} of 2.0. The cell suspension was then aliquoted and treated with 20 μM of probe **12** and reference **10** for 12 h. The cells were then washed twice with 1× PBS buffer. Fluorescence of the bacterial solutions was measured ($\lambda_{ex}/\lambda_{em}$ = 355/550 nm).

Fluorescence lifetime imaging of live *E. coli*. *E.coli* (ATCC25922) cells cultured overnight in LB medium were harvested and washed twice with PBS buffer solution. The washed cells were resuspended in 50 mM Tris-HCl (pH 7.4) buffer with an OD600 of 0.5 - 0.7. Then 500 μL aliquots were treated with 20 μM of probe **12**. After incubation at 37 °C for 4 h, a drop of the suspension was added into an 8-well chamber followed by covering with agarose pads. Fluorescence lifetime images were acquired by an ISS Q2 confocal laser scanning system coupled to a Nikon TE2000 microscope with the 60×/1.2 NA WI objective lens. The excitation wavelength of the probe **12** was 375 nm (5000 Hz repetition rate), fluorescence emission and lifetime signals were collected through a 641/75 nm long-pass edge filter.

Synthetic Procedures

Methyl 2-bromo-4-nitrobenzoate

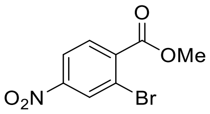

2-Bromo-4-nitrobenzoic acid (**1**, 500 mg, 2.03 mmol) was dissolved in MeOH (10 ml) and thionyl chloride (295 µl, 4.06 mmol) was added. The mixture was stirred at 65°C for 18 h, allowed to cool down to r.t. and neutralized by addition of aq. sat. $NaHCO_3$. The aq. phase was extracted with EtOAc (3x) and the combined org. layers were dried over $MgSO_4$, filtered and the solvent was evaporated to provide 483 mg (92%) as methyl 2-bromo-4-nitrobenzoate as colorless solid.

^{1}H-NMR (300 MHz, $CDCl_3$): δ = 3.99 (s, 3 H, CH_3), 7.92 (d, J = 8.5 Hz, 1 H, 6-H), 8.21 (dd, J = 8.5, 2.2 Hz, 1 H, 5-H), 8.52 (d, J = 2.2 Hz, 1 H, 3-H) ppm. Data matches literature.[2]

Methyl 4-amino-2-bromobenzoate (2)

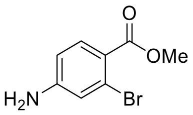

Methyl 2-bromo-4-nitrobenzoate (364 mg, 1.40 mmol) was dissolved in ethanol/ethyl acetate (20+10 ml) and tin chloride dihydrate (1.58 g, 7.00 mmol) was added. The solution was heated to 85°C for 4 h, then was cooled down to r.t. and poured into water (30 ml). Sat. aq. $NaHCO_3$ (10 ml) was added dropwise and the slurry was extracted two times with ethyl acetate. The org. layers were washed with brine, dried over $MgSO_4$, and filtered. Evaporation of the solvents yielded 304 mg (94%) methyl 4-amino-2-bromobenzoate (**2**) as yellow resin.

^{1}H-NMR (300 MHz, $CDCl_3$): δ = 3.86 (s, 3 H, CH_3), 6.56 (dd, J = 8.5, 2.3 Hz, 1 H, 5-H), 6.93 (d, J = 2.3 Hz, 1 H, 3-H), 7.76 (d, J = 8.5 Hz, 1 H, 6-H) ppm. **^{13}C-NMR** (75 MHz, $CDCl_3$): δ = 52.0 (CH_3), 112.9 (C-5), 119.8 (C-1), 119.9 (C-3), 124.3 (C-2), 133.8 (C-6), 150.6 (C-4), 166.1 (CO_2Me) ppm. **HRMS** (pos. ESI-TOF): *m/z* calculated for $C_8H_9BrNO_2$ $[M+H]^+$ 229.9811, found 229.9818.

(4-Amino-2-bromophenyl)methanol

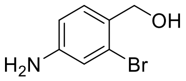

A solution of methyl 4-amino-2-bromobenzoate (**2**, 500 mg, 2.17 mmol) in THF (7 ml) was added dropwise to a solution of lithiumtetrahydroaluminate (3.26 ml, 6.52 mmol, 2 M in THF) in THF (7 ml) at 0°C. The reaction was stirred for 3 h while cooling. Water was added carefully, then 2 M NaOH was added and the mixture was stirred until a fine precipitate had formed. The mixture was filtered over celite, ethyl acetate was added and the org. layer was separated. The aq. phase was extracted with ethyl acetate and the combined org. layers were washed with brine, dried over $MgSO_4$ and filtered. The crude was purified by flash chromatography (SiO_2, cHex/EA 3:1 - 1:1) to provide 299 mg (67%) (4-amino-2-bromophenyl)methanol as colorless crystalline solid.

^{1}H-NMR (300 MHz, CD_3CN): δ = 3.02 (t, J = 5.9 Hz, 1 H, OH), 4.26 (s, 2 H, NH_2), 4.47 (d, J = 5.7, 2 H, CH_2), 6.61 (dd, J = 8.2, 2.3 Hz, 1 H, 5-H), 6.84 (d, J = 2.3 Hz, 1 H, 3-H), 7.16 (d, J = 8.2 Hz, 1 H, 6-H) ppm. **^{13}C-NMR** (75 MHz, CD_3CN): δ = 64.2 (CH_2), 114.5 (C-5), 118.4 (C-3), 123.9 (C-2), 129.5 (C-1), 131.0 (C-6), 149.6 (C-4) ppm. **HRMS** (pos. ESI-TOF): *m/z* calculated for C_7H_9BrNO $[M+H^+]$ 201.9862, found 201.9869.

***tert*-Butyl (3-bromo-4-(hydroxymethyl)phenyl)carbamate (3)**

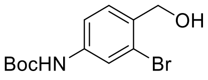

(4-Amino-2-bromophenyl)methanol (45 mg, 0.22 mmol) was dissolved in THF (5 ml) and DIPEA (58 µl, 0.34 mmol) and boc anhydride (97 mg, 0.45 mmol) was added. The reaction was stirred at r.t. for 4 d. It was diluted with water and the solution was extracted with ethyl acetate (2x) dried over $MgSO_4$ and filtered. Purification of the crude by flash chromatography (cHex/EA 3:1) yielded 55 mg (82%) of the title compound (**3**) as colorless oil.

^{1}H-NMR (300 MHz, $CDCl_3$): *δ* = 1.51 .(s, 9 H, $C(CH_3)_3$), 4.68 (s, 2 H, C*H*$_2$OH), 6.52 (s, 1 H, N*H*Boc), 7.21 (dd, *J* = 8.3, 2.2 Hz, 1 H, 6-H), 7.35 (d, *J* = 8.3 Hz, 1 H, 5-H), 7.73 (d, *J* = 2.2 Hz, 1 H, 2-H) ppm. **^{13}C-NMR** (75 MHz, $CDCl_3$): *δ* = 28.4 (C(*C*H$_3$)$_3$), 64.9 (CH_2OH), 81.2 (*C*(CH$_3$)$_3$), 117.5 (C-6), 122.3 (C-2), 123.2 (C-3), 129.6 (C-5), 134.2 (C-4), 139.1 (C-1), 152.5 (CONH) ppm. **HRMS** (pos. ESI-TOF): *m/z* calculated for $C_{12}H_{16}BrNO_3Na$ $[M+Na]^+$ 326.0186, found 326.0181.

tert-Butyl (3-bromo-4-formylphenyl)carbamate (4)

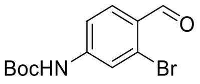

tert-Butyl (3-bromo-4-(hydroxyl-methyl)phenyl)carbamate (**3**, 70 mg, 0.23 mmol) was dissolved in dichloromethane (10 ml) and Dess-Martin periodinane (147 mg, 0.35 mmol) was added. The reaction was stirred at r.t. for 18 h, diluted with ethyl acetate and washed with sat. aq. $NaHCO_3$, water, and brine. The org. layer was dried over $MgSO_4$ and filtered. Purification of the crude by flash chromatography (SiO_2, cHex/EA = 4:1) yielded 52 mg (76%) *tert*-butyl (3-bromo-4-formylphenyl)carbamate (**4**) as colorless solid.

^{1}H-NMR (300 MHz, $CDCl_3$): *δ* = 1.52 (s, 9 H, C(C*H*$_3$)$_3$), 6.94 (s, 1 H, NH), 7.28 (ddd, *J* = 8.5, 2.1, 0.8 Hz, 1 H, 6-H), 7.84 (d, *J* = 8.5 Hz, 1 H, 5-H), 7.92 (d, *J* = 2.1 Hz, 1 H, 2-H), 10.21 (d, *J* = 0.8 Hz, 1 H, CHO) ppm. **^{13}C-NMR** (75 MHz, $CDCl_3$): *δ* = 28.3 (C(*C*H$_3$)$_3$), 82.1 (*C*(CH$_3$)$_3$), 117.0 (C-6), 122.0 (C-2), 128.3 (C-4), 128.7 (C-3), 130.8 (C-5), 144.9 (C-1), 151.9 (CONH), 191.0 (CHO) ppm. **HRMS** (pos. ESI-TOF): *m/z* calculated for $C_{12}H_{15}BrNO_3$ $[M+H]^+$ 302.0210, found 302.0215.

Di-*tert*-butyl (4-formyl-1,3-phenylene)dicarbamate

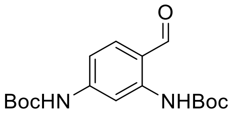

In a screw-cap vial, *tert*-Butyl (3-bromo-4-formylphenyl)carbamate (**4**, 54 mg, 0.18 mmol), caesium carbonate (88 mg, 0.27 mmol) and *tert*-butyl carbamate (28 mg, 0.23 mmol) were dissolved in dioxane and the solvent was deoxygenated by bubbling nitrogen through the solution for 15 min. Xantphos Pd G3 (6 mg, 0.006 mmol) was added and the mixture was heated to 100°C for 18 h. After cooling down to r.t. it was partitioned between DCM and water and the org. layer was separated, dried over $MgSO_4$ and filtered. Flash chromatography (SiO_2, cHex/EA = 95:5) provided 42 mg (69%) of the title compound as colorless crystalline solid.

^{1}H-NMR (300 MHz, $CDCl_3$): *δ* = 1.48, 1.50 (2 s, 18 H, $C(CH_3)_3$), 6.83 (s, 1 H, NH), 7.53 (s, 2 H, 5,6-H), 8.16 (s, 1 H, 3-H), 9.74 (s, 1 H, CHO), 10.51 (s, 1 H, NH) ppm. **^{13}C-NMR** (75 MHz, $CDCl_3$): *δ* = 28.4, 28.4 (C(*C*H$_3$)$_3$), 81.2, 81.7 (*C*(CH$_3$)$_3$), 106.4 (C-3), 110.8 (C-5), 117.0 (C-1), 138.1 (C-6), 143.1 (C-2), 145.7 (C-4), 152.0, 153.1 (NHCO), 193.4 (CHO) ppm. **HRMS** (pos. ESI-TOF): *m/z* calculated for $C_{17}H_{24}N_2O_5Na$ $[M+Na]^+$ 359.1577, found 359.1591.

Diethyl 2-(2,4-bis((tert-butoxycarbonyl)amino)benzylidene)malonate (5)

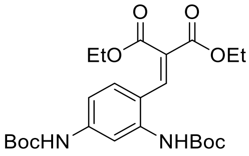

This compound was prepared following a procedure from Lehnert.[3] A solution of titanium tetrachloride (16 µl, 0.14 mmol) in THF (5 ml) was cooled to 0°C. Di-*tert*-butyl (4-formyl-1,3-phenylene)dicarbamate (24 mg, 0.07 mmol) and diethyl malonate (13 µl, 0.09 mmol) in THF (5 ml) were added, then pyridine (23 µl, 0.28 mmol) was added dropwise. The solution was stirred for 4 h while warming up to r.t. Water was added and the mixture was extracted with ethyl acetate (3x). The combined org. layers were dried over $MgSO_4$, filtered and the crude was purified by flash chromatography (cHex/EA = 4:1) to yield 11 mg (32%) of the title compound (**5**) as colorless oil.

^{1}H-NMR (300 MHz, $CDCl_3$): *δ* = 1.22 (t, *J* = 7.1 Hz, 3 H, CH_3), 1.33 (t, *J* = 7.1 Hz, 3 H, CH_3), 1.50 (s, 9 H, $C(CH_3)_3$), 1.51 (s, 9 H, $C(CH_3)_3$), 4.24 (q, *J* = 7.1 Hz, 2 H, CH_2CH_3), 4.30 (q, *J* = 7.1 Hz, 2 H, CH_2CH_3), 6.45, 6.66 (2 s, 2 H, NHCO), 7.24-7.33 (m, 2 H, 5-H, 6-H), 7.74 (s, 1 H, CH), 7.78 (m_c, 1 H, 3-H) ppm. **^{13}C-NMR** (75 MHz, $CDCl_3$): *δ* = 14.1, 14.3 (CH_3), 28.4, 28.4 ($C(CH_3)_3$), 61.8, 61.9 (CH_2CH_3), 81.1, 81.5 ($C(CH_3)_3$), 110.7 (C-3) 113.5 (C-5), 118.6 (C-1), 127.9 (C-2), 129.6 (C-6), 137.7 (CH), 137.9 (*C*-CH), 141.3 (C-4), 152.4, 152.6 (CONH), 164.2, 166.3 (COOEt). **HRMS** (pos. ESI-TOF): *m/z* calculated for $C_{24}H_{35}N_2O_8$ $[M+H]^+$ 479.2388, found 479.2396.

Ethyl 7-amino-2-oxo-1,2-dihydroquinoline-3-carboxylate (6)

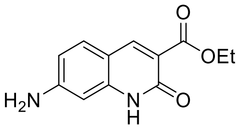

Diethyl 2-(2,4-bis((tert-butoxycarbonyl)amino)benzylidene)malonate (**5**, 9 mg, 0.02 mmol) was dissolved in DCM/TFA (5 ml, 1:1) and the solution was stirred at r.t. for 30 min. The solvent was evaporated and the residue was suspended in H_2O and lyophilized to yield 7 mg (quant.) ethyl 7-amino-2-oxo-1,2-dihydroquinoline-3-carboxylate (**6**) as pale yellow powder.

^{1}H-NMR (300 MHz, CD_3OD): *δ* = 1.36 (t, *J* =7.1 Hz, 3 H, CH_3), 4.32 (q, *J* = 7.1 Hz, 2 H, CH_2), 6.40 (s, 1 H, 8-H), 6.64 (d, *J* = 8.7 Hz, 1 H, 6-H), 7.43 (d, *J* = 8.7 Hz, 1 H, 5-H), 8.48 (s, 1 H, 4-H) ppm. **^{13}C-NMR** (75 MHz, CD_3OD): *δ* = 14.6 (CH_3), 62.0 (CH_2), 96.5 (C-8), 111.9 (C-4-*C*-C-5), 114.4 (C-6), 132.6 (C-5), 144.6 (C-8-*C*-N), 147.7 (C-4), 156.4 (C-7), 163.2 (NHCO), 167.1 (NHCO) ppm. **HRMS** (pos. ESI-TOF): *m/z* calculated for $C_{12}H_{13}N_2O_3$ $[M+H]^+$ 233.0921, found 233.0917.

***tert*-Butyl (4-nitrobenzyl) (4-formyl-1,3-phenylene)dicarbamate (7)**

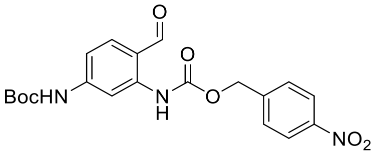

4-Nitrobenzyl carbamate was prepared as described previously.[4]

tert-Butyl (3-bromo-4-formylphenyl)carbamate (**4**, 200 mg, 0.67 mmol), caesium carbonate (326 mg, 1.0 mmol) and 4-nitrobenzyl carbamate (170 mg, 0.87 mmol) were added in a screw-cap vial, dioxane (5 ml) was added and the solution was deoxygenated by bubbling argon through the solution for 15 min. Subsequently, Xantphos Pd G3 (22 mg, 23 µmol) was added and the reaction was heated to 100°C for 5 h. Water and ethyl acetate were added and the org. layer was separated. The aq. phase was extracted once with ethyl acetate and the combined org. layers were dried over Na_2SO_4, filtered and the crude was purified by flash chromatography (Hept/EA = 8:2 - 6:4) to yield 258 mg (88%) *tert*-butyl (4-nitrobenzyl) (4-formyl-1,3-phenylene)dicarbamate (**7**) as colorless solid.

^{1}H-NMR (600 MHz, DMSO-D_6): *δ* = 1.48 (s, 9 H, $C(CH_3)_3$), 5.35 (s, 2 H, CH_2), 7.29 (dd, *J* = 8.6, 2.0 Hz, 1 H, 5-H), 7.67-7.73 (m, 2 H, 2',6'-H), 7.75 (d, *J* = 8.6 Hz, 1 H, 6-H), 8.24-8.29 (m, 2 H, 3',5'-H), 8.51 (d, *J* = 2.0 Hz, 1 H, 3-H), 9.78 (s, 1 H, NH), 9.99 (s, 1 H, NH), 10.82 (s, 1 H, CHO) ppm. **^{13}C-NMR** (150 MHz, DMSO-D_6): *δ* = 28.0 ($C(CH_3)_3$), 65.2 (CH_2), 80.1 ($C(CH_3)_3$), 106.4 (C-3), 112.0 (C-5), 116.8 (C-1), 123.7 (C-3',5'), 128.6 (C-2',6'), 136.7 (C-6), 141.4 (C-2), 144.0 (C-1'), 146.6 (C-4), 147.2 (C-4'), 152.3 (NHCO), 152.4 (NHCO), 193.9 (*C*HO) ppm. **HRMS** (neg. ESI-TOF): *m/z* calculated for $C_{20}H_{20}N_3O_7$ $[M-H]^-$ 414.1307, found 414.1323.

Boc-protected caged antenna

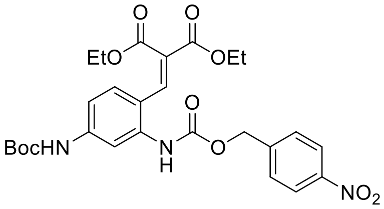

A solution of titanium tetrachloride (1.16 ml, 1.16 mmol, 1 M in DCM) in tetrahydrofuran (2 ml) was cooled to 0°C. *tert*-Butyl (4-nitrobenzyl) (4-formyl-1,3-phenylene)dicarbamate (**7**, 240 mg, 0.58 mmol) in tetrahydrofuran (2 ml) and diethyl malonate (106 µl, 0.69 mmol) were added, then pyridine (186 µl, 2.31 mmol) was added dropwise. The solution was allowed to warm up to r.t. and stirred for 18 h. 2 M aq. $KHCO_3$ and ethyl acetate were added and the layers were separated. The aq. phase was extracted once with ethyl acetate and the combined org. phases were washed with brine, dried over Na_2SO_4 and filtered. Purification of the crude by flash chromatography (Hept/EA = 7:3 - 1:1) yielded 261 mg (81%) of the title compound as light yellow solid.

^{1}H-NMR (600 MHz, DMSO-D$_6$): δ = 1.19 (t, J = 7.1 Hz, 3 H, CH_3), 1.22 (t, J = 7.1 Hz, 3 H, CH_3), 1.47 (s, 9 H, $C(CH_3)_3$), 4.21 (q, J = 7.1 Hz, 2 H, CH_2CH_3), 4.23 (q, J = 7.1 Hz, 2 H, CH_2CH_3), 5.30 (s, 2 H, CH_2), 7.21-7.25 (m, 1 H, 6-H), 7.26-7.29 (m, 1 H, 5-H), 7.68 (d, J = 8.7 Hz, 2 H, 2',6'-H), 7.71 (d, J = 1.4 Hz, 1 H, 3 H), 7.73 (s, 1 H, CH=C), 8.26 (d, J = 8.7 Hz, 2 H, 3',5'-H), 9.67, 9.71 (2 s, 2 H, NHCO) ppm. **^{13}C-NMR** (150 MHz, DMSO-D$_6$): δ = 13.4 (CH_3), 13.9 (CH_3), 28.0 ($C(CH_3)_3$), 61.2 (CH_2CH_3), 61.2 (CH_2CH_3), 64.8 (CH_2Ar), 79.7 ($C(CH_3)_3$), 115.0 (C-5), 120.6 (C-1), 123.6 (C-3',5'), 124.2 (CH=*C*), 128.3 (C-2',6'), 128.4 (C-6), 137.8 (*C*H=C), 138.2 (C-2), 142.4 (C-4), 144.6 (C-1'), 147.0 (C-4'), 152.5 (NHCO), 153.9 (NHCO), 163.8 (CO_2Et), 166.0 (CO_2Et) ppm. **HRMS** (pos. ESI-TOF): *m/z* calculated for $C_{27}H_{32}N_3O_{10}$ $[M+H]^+$ 558.2082, found 558.2080.

Caged antenna (8)

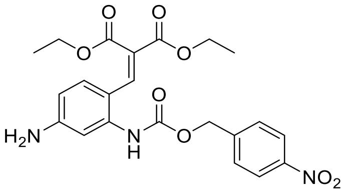

Boc-protected antenna precursor (349 mg, 0.63 mmol) was dissolved in DCM/TFA (10 ml, 1:1) and the solution was stirred for 30 min. The solvents were evaporated and the residue was suspended in water/acetonitrile and lyophilized to obtain 183 mg (64%) of the title compound (**8**) as pale yellow powder which was directly used for the next step without further purification.

DOTA antenna conjugate (9)

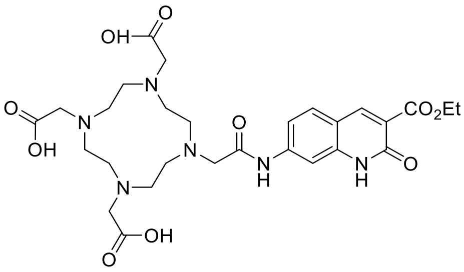

Ethyl 7-aminoquinolin-2-one-3-carboxylate (**6**, 73 mg, 0.16 mmol) was dissolved in DMF (5 ml) and the solution was cooled to 0°C. Bromoacetyl bromide (20 µl, 0.22 mmol) and potassium carbonate (48 mg, 0.34 mmol) were added successively and the reaction was stirred at 0°C for 2 h. The mixture was diluted with dichloromethane (20 ml) and the org. layer was washed with water, dried over $MgSO_4$ and filtered. The solvents were evaporated, the residue was dissolved in DMF (5 ml) and *tri*-tert-butyl 2,2',2''-(1,4,7,10-tetraazacyclododecane-1,4,7-triyl)triacetate (115 mg, 0.22 mmol) and potassium carbonate (48 mg, 0.34 mmol) were added and the mixture was stirred at r.t. for 18 h. The solids were removed by filtration over celite and the solvents were evaporated. The crude was dissolved in TFA/DCM (5 ml, 1:1) and the solution was stirred for 18 h at r.t. Evaporation of the solvents and purification by HPLC (**Method A**) yielded 15 mg (14% over 3 steps) of the title compound (**9**) as colorless powder after lyophilization.

^{1}H-NMR (600 MHz, MeOD): δ = 1.41 (t, J = 7.1 Hz, 3 H, CH_3), 3.30-4.35 (m, 24 H, NCH_2CH_2, NCH_2CO), 4.37 (q, J = 7.1 Hz, 2 H, CH_2CH_3), 6.89 (bs, 1 H, 6-H), 7.39 (d, J = 8.5 Hz, 1 H, 5-H), 7.81 (s, 1 H, 8-H), 8.33 (s, 1 H, 4-H) ppm. **^{13}C-NMR** (150 MHz, MeOD): δ = 14.7 (CH_3), 50.1, 52.4 (NCH_2CH_2), 54.1, 55.8, 56.2 (NCH_2CO), 62.2 (CH_2CH_3), 105.3 (C-8), 115.7 (C-3), 116.1 (C-6), 118.2 (q, J = 290 Hz, CF_3COO), 120.1 (C-4-*C*-C-5), 131.1 (C-5), 142.3 (C-8-*C*), 144.3 (C-7), 146.4 (C-4), 161.8 (CONH), 162.8 (q, J = 34 Hz, CF_3COO), 165.8 (COOEt), 170.6 (CH_2CONH), 173.9 (COOH) ppm. **HRMS** (pos. ESI-TOF): *m/z* calculated for $C_{28}H_{39}N_6O_{10}$ $[M+H]^+$ 619.2722, found 619.2723.

DOTA caged antenna conjugate (11)

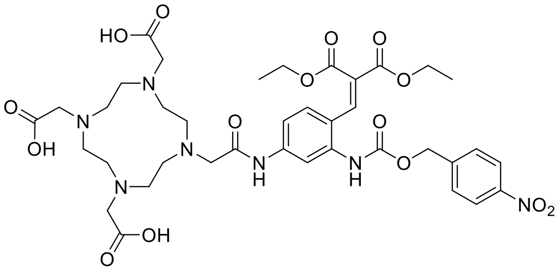

Caged antenna (**8**, 73 mg, 0.16 mmol) was dissolved in acetonitrile (5 ml) and the solution was cooled to 0°C. Potassium carbonate (44 mg, 0.32 mmol) and bromoacetyl bromide (18 µl, 0.21 mmol) were added successively and the reaction was stirred at 0°C for 2 h. The mixture was diluted with dichloromethane (20 ml) and the org. layer was washed with water, dried over $MgSO_4$ and filtered. The solvents were evaporated to furnish 93 mg (quant.) of a yellow oil which was used without further purification. It was dissolved in acetonitrile (5 ml) and *tri*-tert-butyl 2,2',2''-(1,4,7,10-tetraazacyclododecane-1,4,7-triyl)triacetate (91 mg, 0.18 mmol) and potassium carbonate (34 mg, 0.24 mmol) were added and the mixture was stirred at r.t. for 18 h. The solids were removed by filtration over celite and the solvents were evaporated to yield 152 mg of the *tert*-butyl protected DOTA derivative as yellow oil. It was dissolved in 4 N HCl in dioxane (5 ml) and the solution was stirred at r.t. for 18 h. The solvents were evaporated and the residue was purified by HPLC (**Method B**) to yield 64 mg (47% over 3 steps) of the title compound (**11**) as white powder.

^{1}H-NMR (300 MHz, MeOD): δ = 1.24 (t, J = 7.1 Hz, 3 H, CH_3), 1.29 (t, J = 7.1 Hz, 3 H, CH_3), 2.85-3.55 (m, 16 H, NCH_2CH_2), 3.65-4.07 (m, 8 H, NCH_2CO), 4.26 (q, J = 7.1 Hz, 4 H, CH_2CH_3), 5.29 (s, 2 H, CH_2Ar), 7.34 (d, J = 8.5 Hz, 1 H, 5-H), 7.58 (d, J = 8.5 Hz, 1 H, 6-H), 7.63 (d, J = 8.4 Hz, 2 H, 2',6'-H), 7.69 (bs, 1 H, 3-H), 7.77 (s, 1 H, $CHC(CO_2Et)_2$), 8.23 (d, J = 8.4 Hz, 2 H, 3',5'-H) ppm. **^{13}C-NMR** (75 MHz, MeOD): δ = 14.3, 14.5 (CH_3), 50.8, 51.5 ($N(CH_2CH_2)$), 55.6, 56.1 (NCH_2CO), 62.8, 62.9 (CH_2CH_3), 66.6 (CH_2Ar), 116.9 (C-3), 117.8 (C-6), 118.2 (q, $^{1}J_{CF}$ = 293 Hz, CF_3CO_2H), 124.7 (C-3',5'), 127.4 ($C(CO_2Et)_2$), 129.3 (C-2',6'), 130.3 (C-5), 139.1 (C-1), 139.5 ($CHC(CO_2Et)_2$), 142.4 (C-4), 145.7 (C-1'), 148.9 (C-4'), 156.3 ($NHCO_2$), 162.8 (q, $^{3}J_{CF}$ = 35 Hz, CF_3CO_2H), 165.6, 168.1 (CO_2Et) ppm. CO_2H and CONH are not really visible presumably due to different conformational isomers and the resulting decrease in peak intensity. **HRMS** (pos. ESI-TOF): *m/z* calculated for $C_{38}H_{50}N_7O_{15}$ $[M+H]^+$ 844.3359, found 844.3372.

General procedure for terbium chelation

DOTA conjugate (1 eq.) was dissolved in ethanol (0.01 M) and terbium trichloride (3 eq.) was added. Water was added dropwise until full dissolution of the components, then the solution was heated to 45°C and stirred for 24 h. Water (3 ml) was added, ethanol was evaporated and the aqueous solution was purified by HPLC.

Tb - DOTA antenna conjugate (10)

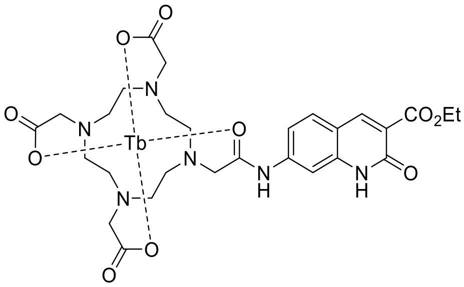

HPLC **Method A**, 8 mg (50%). **HRMS** (pos. ESI-TOF): *m/z* calculated for $C_{28}H_{36}N_6O_{10}Tb$ $[M+H]^+$ 775.1741, found 775.1758.

Tb - DOTA caged antenna conjugate (12)

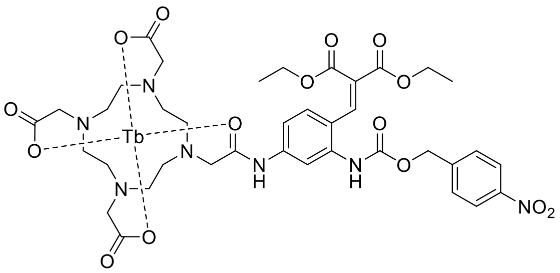

HPLC **Method C**, 24 mg (40%). **HRMS** (pos. ESI-TOF): *m/z* calculated for $C_{38}H_{47}N_7O_{15}Tb$ $[M+H]^+$ 1000.2378, found 1000.2382.

Probe Characterization

Absorption/Excitation/Emission Spectra

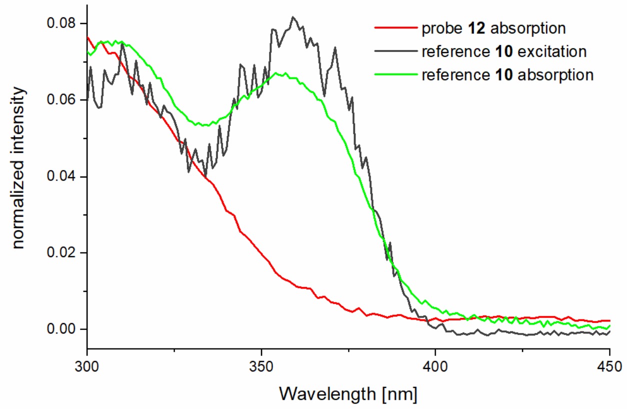

Figure S1. Absorption spectra of reference **10** and probe **12** and excitation spectrum of reference **10** (20 µM) in PBS buffer pH 7.4. Excitation monitored at 545 nm.

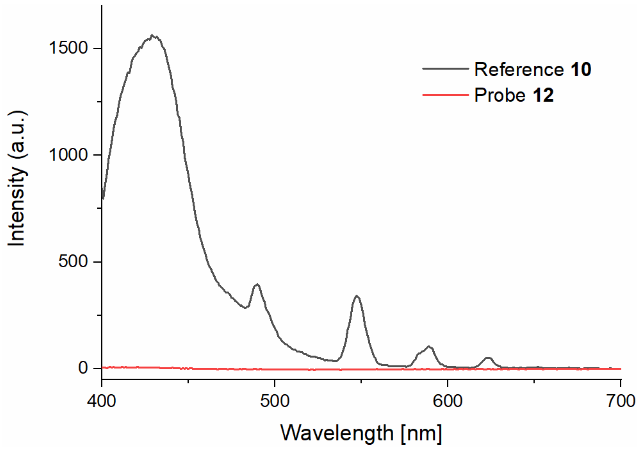

Figure S2. Steady-state fluorescence spectra of reference **10** and probe **12** (20 µM) in PBS pH 7.4, λ_{ex} = 355 nm.

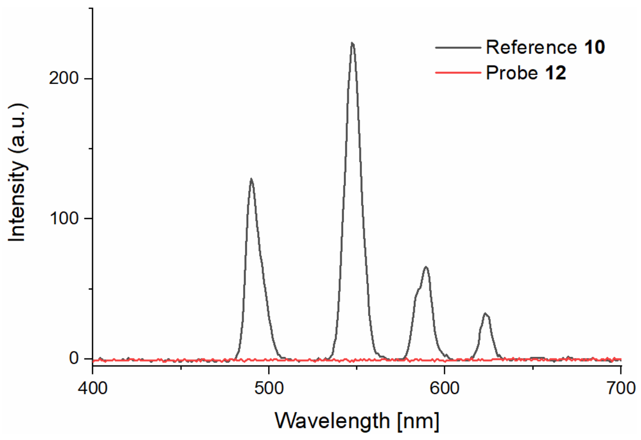

Figure S3. Time-gated fluorescence spectra reference **10** and probe **12** (20 µM) in PBS pH 7.4 (50 µs delay), λ_{ex} = 355 nm.

Lifetime Measurements

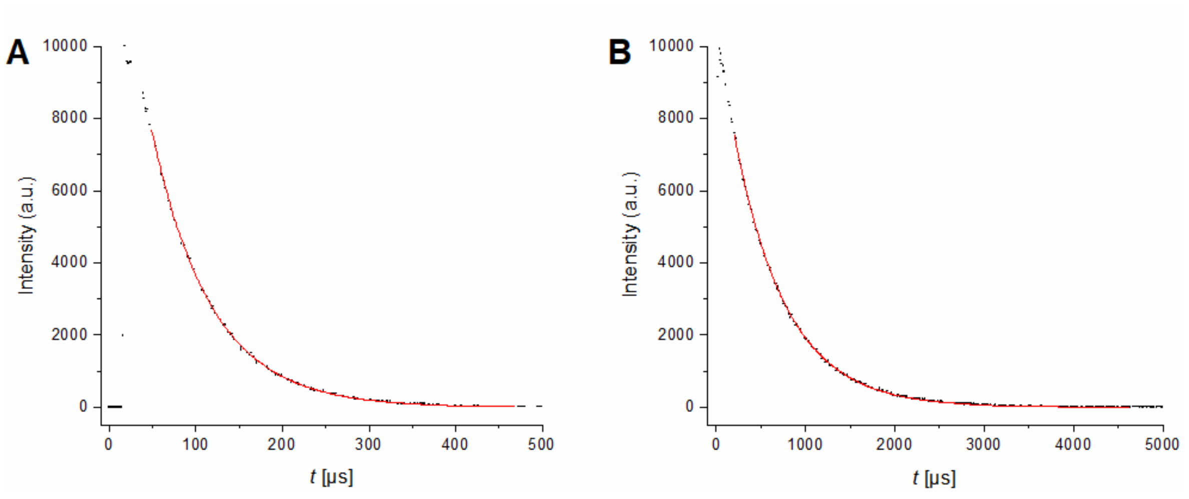

Figure S4: Photoluminescence intensity decays of reference **10**, 20 μM in TRIS-HCl buffer (pH 7.4) at r.t. (black dots) and monoexponential decay fits (red lines, R^2 = 0.9996). **A:** under air environment; **B:** oxygen-free. λ_{ex} = 355 nm, λ_{em} = 550 nm.

Table S1. Luminescent lifetimes of compounds **10** and **12** obtained from single exponential fit of the intensity decays.

	τ [μs][a]	τ [μs][b]
reference **10**	68	580
probe **12**[c]	n.a.	n.a.

[a]Under air environment; [b]oxygen-free; [c]probe **12** is non-fluorescent.

Determination of kinetic parameters

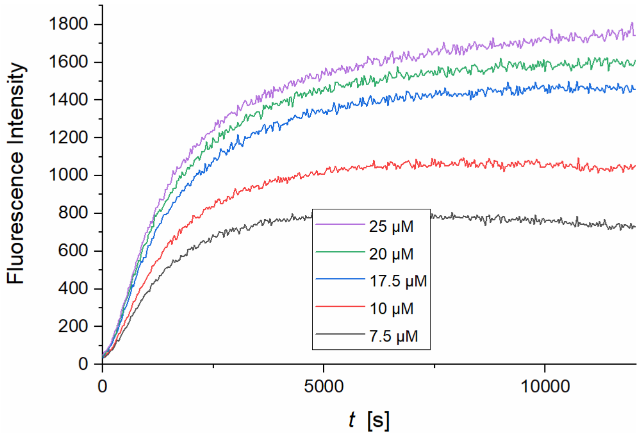

Figure S5. Fluorescence intensity of probe **12** (7.5, 10.0, 15.0, 17.5, 20.0, 25.0 µM) at 550 nm upon incubation with nitroreductase (1 µg/ml). The measurements were performed at 37 °C in 0.05 M Tris buffer (pH 7.4) in the presence of 50 µM NADH. λ_{ex} = 355 nm. Results are representative of two independent experiments.

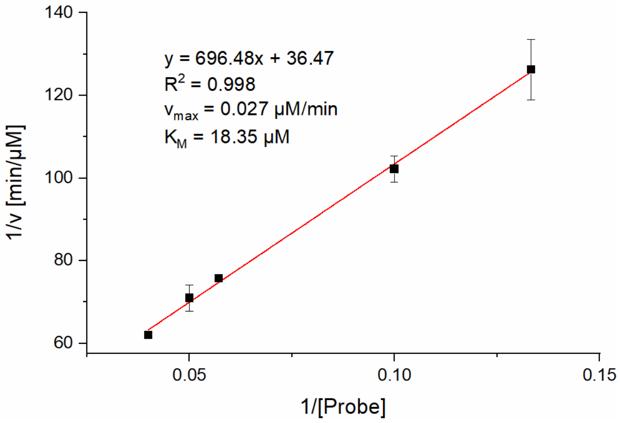

Figure S6. Lineweaver-Burk plot for the nitroreductase-mediated activation of probe **12** derived from the Michaelis-Menten equation. The Michaelis-Menten equation is described as: $v = v_{max}$[probe]/(Km + [probe]), where v is the reaction rate, [Probe] is the probe concentration, and K_M is the Michaelis-Menten constant. Conditions: 1 µg/mL NTR, 50 µM NADH, 7.5-25 µM of the probe, $\lambda_{ex}/\lambda_{em}$ = 355/550 nm. Points are fitted using a linear regression model (correlation coefficient: R^2= 0.998). Results are representative of two independent experiments.

LC/MS activation studies

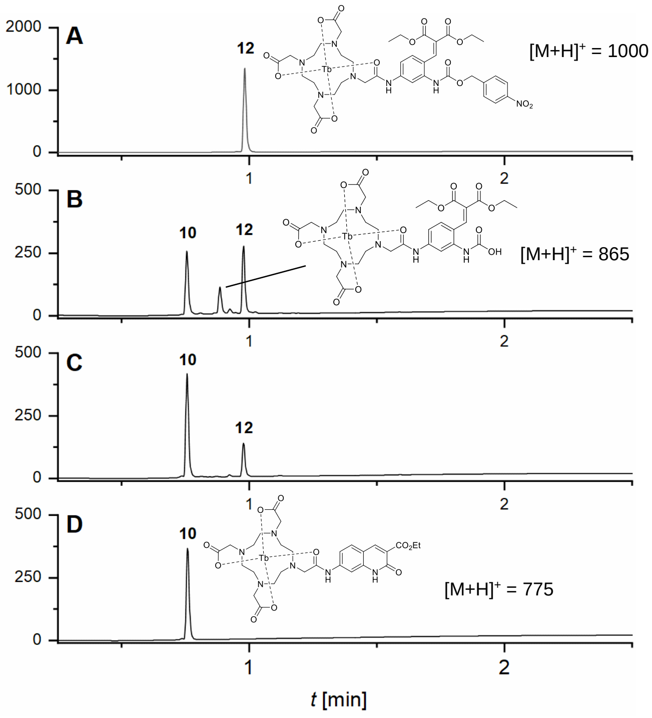

Figure S7. LC/MS activation studies with probe **12 (**100 µM**)** and nitroreductase (30 µg/ml) in presence of NADH (500 µM). **A.** probe **12** + NADH (control); **B.** probe **12** + NADH + nitroreductase (2 h); **C.** probe **12** + NADH + nitroreductase (4.5 h); **D.** reference **10** (control).

Figure 6 shows LC/MS activation studies of probe **12**. **A** and **D** show LC/MS traces of probe **12** and reference **10**, respectively. In presence of nitroreductase and NADH (**B** and **C**, 2 h and 4.5 h incubation, respectively) a time-dependent conversion of probe **12** towards activated probe (reference) **10** is observed. The small peak in **B** corresponds to $[M+H]^+$ = 865, the mass of the carbamic acid fragmentation intermediate.

Effects of pH and temperature

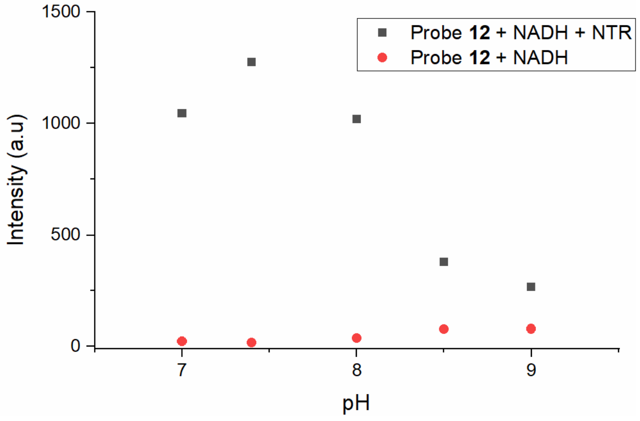

Figure S8. Effect of pH on the fluorescence intensity of probe **12** (20 µM) in absence or presence of nitroreductase (1 µg/mL) and NADH (50 µM) in 0.05 M Tris buffer for 2 h. $\lambda_{ex}/\lambda_{em}$ = 355/550 nm. Results are representative of two independent experiments (error bars not shown when lying within symbol boarders).

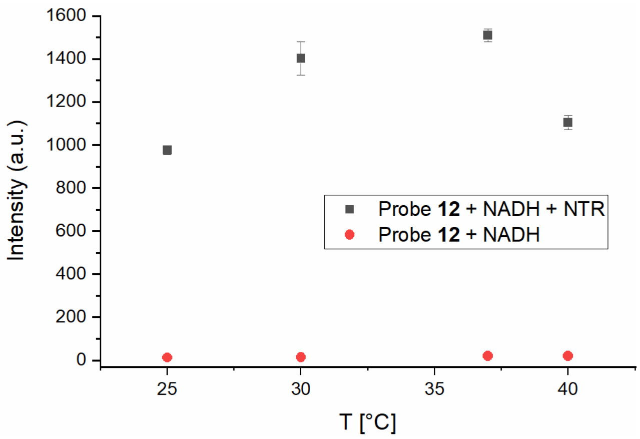

Figure S9. Effect of temperature on the fluorescence intensity of probe **12** (20 µM) in absence or presence of nitroreductase (1 µg/mL) and NADH (50 µM) in 0.05 M Tris buffer for 2 h. $\lambda_{ex}/\lambda_{em}$ = 355/550 nm. Results are representative of two independent experiments (error bars not shown when lying within symbol boarders).

Detection Limit

The detection limit was calculated according to the formula 3σ/k, where σ is the standard deviation of the background noise of 20 µM probe collected at 12 time points and k is the slope of the linear fit obtained from enzyme concentration vs probe emission intensity at 550 nm, 20 µM probe (inset Fig. 1A). With σ = 2.357 and k = 1617 ml/µg, 3σ/k = 4.4 ng/ml.

Inhibition Studies

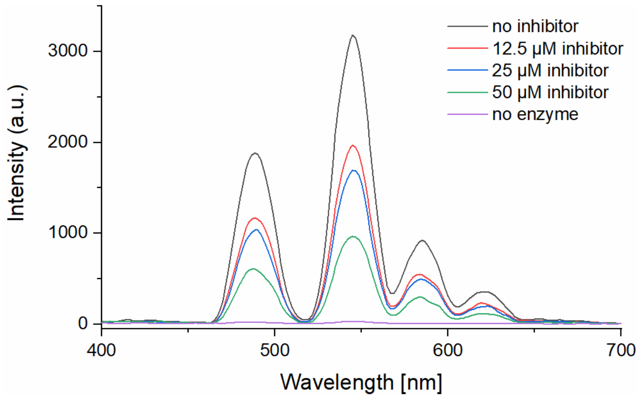

Figure S10. Fluorescence emission spectra of probe **12** (20 µM) with NTR (1 µg/mL) and NADH (50 µM) and with added NTR inhibitor dicoumarin (12.5, 25, 50 µM). *Purple*: probe in pH 7.4 Tris buffer (control). All measurements were carried out after mixing for 2 h at 37 °C. λ_{ex} = 355nm.

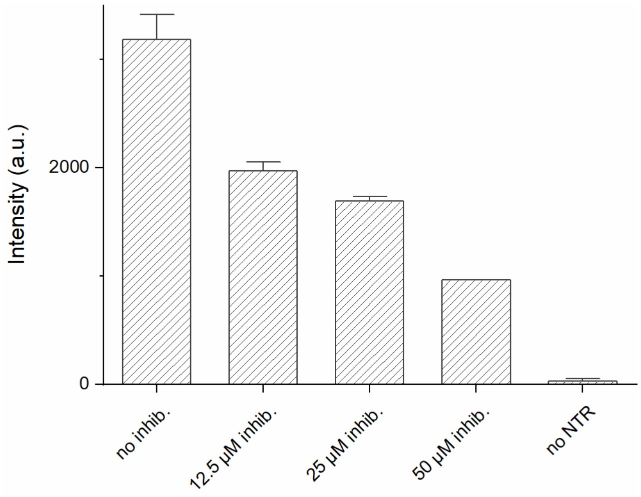

Figure S11. Quantification of emission intensity of probe **12** at 550 nm **i**ncubated with NTR and dicoumarin as shown in Figure 9.

Probe and reference uptake in live bacteria

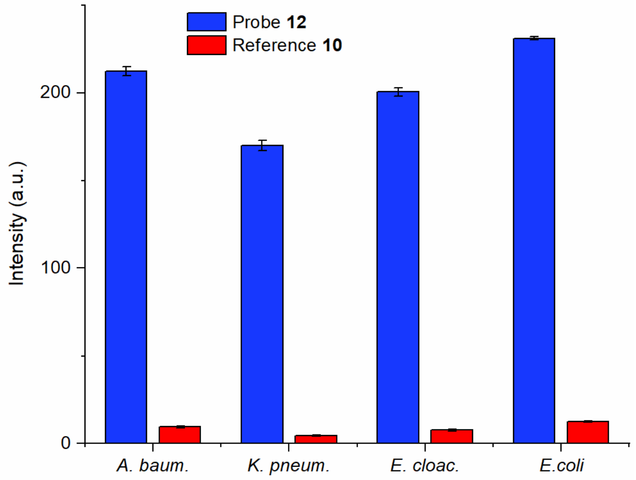

Figure S12. Fluorescence intensities of live *E. coli*, *K. pneumoniae, A. baumannii* and *E. cloacae* cells incubated with 20 µM probe **12** and reference **10** at 37 °C in 0.05 M Tris buffer (pH 7.4) for 12 h. $\lambda_{ex}/\lambda_{em}$ = 355/550 nm, 50 µs delay. Results representative of two independent experiments.

Author Contributions

Marc Nazaré and Hai-Yu Hu conceived the research.
Marc Nazaré and Hai-Yu Hu acquired funding for this project.
Benjamin Brennecke, Qinghua Wang, Hai-Yu Hu and Marc Nazaré designed the research approach.
Benjamin Brennecke, Qinghua Wang and Qingyang Zhang performed experiments, analyzed raw data and worked on probe validation.
Benjamin Brennecke, Qinghua Wang, Hai-Yu Hu and Marc Nazaré analyzed all data generated, and wrote the manuscript.

[1] L. Mangolini, D. Jurbergs, E. Rogojina, U. Kortshagen, *J. Lumin.* **2006**, *121*, 327-334.
[2] P. Wu, J. Zhang, Q. Meng, B. Nare, R. T. Jacobs, H. Zhou, *Eur. J. Med. Chem.* **2014**, *81*, 59-75.
[3] W. Lehnert, *Tetrahedron* **1973**, *29*, 635-638.
[4] K. N. Hearn, T. D. Nalder, R. P. Cox, H. D. Maynard, T. D. M. Bell, F. M. Pfeffer, T. D. Ashton, *Chem. Commun.* **2017**, *53*, 12298-12301.
